# Considerations on allergic rhinitis associated with the degree of pulmonary involvement due to COVID-19 in patients from a Peruvian general hospital

**DOI:** 10.17843/rpmesp.2023.402.12855

**Published:** 2023-06-30

**Authors:** Valeria N. Mendoza-Vera, María J. Huamani-Morales, Dean J. Pineda-Llamocca

**Affiliations:** 1 Universidad Ricardo Palma, Lima, Peru. Universidad Ricardo Palma Universidad Ricardo Palma Lima Peru

To the editor. We interestingly reviewed the published article by García-Gallo *et al*. ^1^ and consider that it is important to mention some aspects that are not clearly described in the limitations.

There are risk factors that could influence the degree of pulmonary involvement by COVID-19 such as chronic kidney disease, chronic liver disease, other immunodeficiencies, chronic neurological disorders, sickle cell disease and severe obesity, which are also considered to be risk factors for severe forms of COVID-19 ^2^; however, it is unclear how this information is retrieved and included in the “comorbidities” variable in the study. Furthermore, the article does not mention relevant parameters to clinically determine the severity of the disease, such as respiratory rate, oxygen saturation, gasometric PO_2_/FiO_2_ or hemodynamic status, variables that not only influence the severity of the disease, but also play a role in the interpretation of imaging studies ^3^.

Regarding temporality, the study period (2020 and 2021) does not specify the timing of events, or any type of pairing by temporality of the cases in order to reduce bias, which is important because the incidence of severe cases may be higher according to the peak of increase of cases, emergence of new variants, in addition to the ecological and protective effect from immunization against SARS-CoV-2, which began in the country on February 9, 2021, and by the end of the same year it had reached a coverage with 1st, 2nd and 3rd doses of 88%, 79% and 15% of the adult population, respectively ^4^. Furthermore, it is not mentioned whether these variables were included in the regression model, which is adequate to adjust for the effect of other variables. Although the sample by convenience of a single hospital unit cannot be used to obtain generalizable conclusions, it is not known if the allergic rhinitis variable was systematically registered as part of the general standard of care, which would otherwise further increase the risk of bias.

Data regarding either bronchial asthma or allergic rhinitis, as “protective” entities against severe forms of COVID-19 are still controversial. A systematic review and meta-analysis (2020 - 2022) of 294,622 patients by Xu *et al*. suggested that COVID-19 incidence, severity and the risk of hospitalization are lower in people with allergic rhinitis ^5^. However, the study has important design and selection biases, as well as confounding factors, leading to imprecision about the presence or absence of other comorbidities in the studied COVID-19 patients that may have influenced their progression. In contrast, Yang *et al*. conducted a nationwide cohort study in South Korea (2020) on 219,959 patients with allergic rhinitis or bronchial asthma with COVID-19, concluding that these comorbidities lead to an increased risk of susceptibility to SARS-CoV-2 infection and worse clinical outcomes ^6^.

Therefore, the premise that “an allergic state could protect against the severity of COVID-19” should be taken with extreme caution. More scientifically sound studies are needed.
