# Supplementary material for: Considerations on allergic rhinitis associated with the degree of pulmonary involvement due to COVID-19 in patients from a Peruvian general hospital
Source: Rev Peru Med Exp Salud Publica. 2023 Jun 30;40(2):250–1. doi: 10.17843/rpmesp.2023.402.12855 (PMC10953629; doi:10.17843/rpmesp.2023.402.12855)
Supplement: Translation

# Translation: Consideraciones sobre la rinitis alérgica asociada al grado de compromiso pulmonar por COVID-19 en pacientes de un hospital general peruano

**Authors:** Valeria N. Mendoza-Vera, María J. Huamani-Morales, Dean J. Pineda-Llamocca

**Affiliations:** 1 Universidad Ricardo Palma, Lima, Perú.

Sr. Editor, al revisar con interés el artículo recientemente publicado de García-Gallo *et al*. ^1^ es importante mencionar algunos aspectos que no están claramente descritos en las limitaciones.

Existen factores de riesgo que pudieron influenciar en el grado de compromiso pulmonar por COVID-19 como la enfermedad renal crónica, enfermedad hepática crónica, otras inmunodeficiencias, trastornos neurológicos crónicos, drepanocitosis y obesidad severa que son considerados factores de riesgo para formas graves de COVID-19 ^2^, sin embargo, es imprecisa la forma de recuperación exhaustiva de esta información en la variable «comorbilidades» en el artículo. Además, el estudio no menciona parámetros relevantes para determinar clínicamente la gravedad de la enfermedad, como la frecuencia respiratoria, saturación de oxígeno, PO_2_/FiO_2_ gasométrico o el estado hemodinámico, variables que juegan un rol no solo en la gravedad clínica, sino también en la interpretación de los estudios por imágenes ^3^. 

Relacionado a la temporalidad, el período de estudio comprendido entre los años 2020 y 2021 no especifica momentos de los eventos, o algún tipo de pareamiento por temporalidad de casos para reducir el sesgo, debido a que pueden existir momentos de mayor incidencia de casos graves según el pico de aumento de casos, emergencia de nuevas variantes, además del efecto ecológico y de protección relacionado a la inmunización contra el virus SARS-CoV-2, que inició en el país el 09 de febrero del 2021, y que para finales del mismo año se había alcanzado una cobertura con 1°, 2° y 3° dosis mayor del 88%, 79% y 15% de la población adulta; respectivamente ^4^. Además, no se menciona si estas variables fueron tomadas en cuenta en el modelo de regresión, el cual es adecuado para ajustar el efecto de otras variables. Si bien la muestra de una unidad hospitalaria por conveniencia no es extrapolable a conclusiones generalizables, la forma de consignación de la variable rinitis alérgica se desconoce que haya sido tomada sistemáticamente por estándar de cuidado que de no serlo aumentaría más el riesgo de sesgo. 

En relación con las patologías alérgicas, sea asma bronquial o rinitis alérgica, como entidades «protectoras» frente a formas severas de COVID-19, existen datos aún controversiales. Xu *et al*. presentaron una revisión sistemática y meta-análisis (2020 - 2022) de 294, 622 pacientes, sugiriendo que la incidencia de COVID-19, severidad y riesgo de hospitalización son menores en personas con rinitis alérgica ^5^. No obstante, el estudio conlleva importantes sesgos de diseño y selección, al igual que factores de confusión, lo cual conduce a una imprecisión sobre la presencia o ausencia de otras comorbilidades en los pacientes con COVID-19 estudiados que puedan haber influenciado en su progresión. Por el contrario, Yang *et al.* llevaron a cabo un estudio de cohorte nacional en Corea del Sur (2020), sobre 219,959 pacientes con rinitis alérgica o asma bronquial con COVID-19, concluyendo que estas comorbilidades conllevan un mayor riesgo de susceptibilidad a la infección por el virus SARS-CoV-2 y a peores desenlaces clínicos ^6^. 

Por lo antes mencionado, es necesario tomar con suma cautela la premisa que «un estado alérgico podría proteger frente a la severidad de la COVID-19». Estudios de mayor solidez científica serían necesarios.
